# Can Broadening the Kidney Biopsy Criteria Reduce Misleading Diagnoses in Young Patients with End-Stage Renal Diseases? A Survey on the Sicilian Registry of Nephrology, Dialysis, and Transplantation

**DOI:** 10.3390/medicina60122031

**Published:** 2024-12-09

**Authors:** Vincenzo Calabrese, Giovanni Conti, Giulio Geraci, Ligia J. Dominguez, Valeria Cernaro, Maria Teresa Zicarelli, Guido Gembillo, Elisa Longhitano, Domenico Santoro

**Affiliations:** 1 Department of Medicine and Surgery, University of Enna “Kore”, 94100 Enna, Italy; v.calabrese@outlook.it (V.C.); giulio.geraci@unikore.it (G.G.); ligia.dominguez@unikore.it (L.J.D.); 2 Unit of Pediatric Nephrology and Rheumatology, University of Messina, 98125 Messina, Italy; giovanniconti@hotmail.com; 3 Unit of Nephrology and Dialysis, Department of Clinical and Experimental Medicine, A.O.U. “G. Martino”, University of Messina, 98125 Messina, Italy; vcernaro@unime.it (V.C.); guidogembillo@live.it (G.G.); elisa.longhitano@libero.it (E.L.); 4 Department of Medical and Surgical Sciences, University of Catanzaro “Magna Graecia”, 88100 Catanzaro, Italy; mteresa.zicarelli@gmail.com

**Keywords:** dialysis, epidemiology, ESRD, hypertension, kidney diseases

## Abstract

Audits allow analysis of the delivery of care and the prevalence of diseases. This study investigated kidney diseases’ impact on end-stage renal disease (ERSD) in patients younger than 30 years. *Methods*: This analysis is retrospectively conducted on young dialysis-dependent patients included in the Sicilian Registry of Nephrology, Dialysis and Transplantation Participants. It evaluated patients who started dialysis before the age of 30 retrieved in the mentioned registry. The sample was divided into two groups, according to the presence or absence of a specific diagnosis. Baseline features were reported as mean ± sd, median [IQR] and *n* (%). A Student T-test, Mann–Whitney test or Pearson Chi-Square test was performed. Logistic regression analysis detected the association between the variables and the unknown diagnosis, and variables with a *p*-value < 0.2 were added to the multivariate model. ROC curves were drawn including this multivariate prediction. *Results*: In total, 145 patients started dialysis before the age of 30 years. Between patients with and without a diagnosis, the intake of renin–angiotensin–aldosteron system inhibitors (RAASIs) and blood pressure differed enough to be considered as possibly confounding. Logistic regression showed that blood pressure and RAASIs seemed to be related to the unknown diagnosis. ROC curves adjusted for RAASIs and blood pressure provided an AUC = 0.689. *Conclusions:* Although Kidney Disease Improving Global Outcomes (KDIGO) did not include hypertension among biopsy indications, our data suggest that performing renal biopsy in young patients with hypertension and worsening renal function could improve kidney diagnosis management.

## 1. Introduction

Systemic or congenital diseases often cause chronic kidney disease (CKD) in young people with the potential development of end-stage renal disease (ESRD) needing renal replacement therapy [1]. Early diagnosis allows the management of kidney diseases, delaying their progression. According to Sun-Young Ahn [2], the CKD pediatric population presents more comorbidities involving other organs, impairing the cardiovascular system and increasing mortality risk. Furthermore, these patients have a higher risk of ESRD at a young age.

End-stage renal disease (ESRD) is not usual, with an incidence of 8–53 per million person-years, depending on ethnicity [3,4]. Due to the heterogeneous and uncertain safety, the 2024 KDIGO guidelines report the following: “We suggest performing a kidney biopsy as an acceptable, safe, diagnostic test to evaluate cause and guide treatment decisions when clinically appropriate (2D)”. Indeed, no comparative studies evaluating safety have been performed [5]. The same guidelines reduce the biopsy criteria to diseases with rapid a decline in renal function or diseases causing proteinuria.

Various methods can be used to carry out epidemiological investigations: surveillance and descriptive studies can be used to study distribution; analytical studies are used to study determinants [6]. Based on this, even if rarely used, audits and surveys enable the analysis of the delivery of care and the prevalence of diseases at different levels, from a single ward to whole national data [7,8].

Most cases of ERSD in young people seem to be caused by Congenital Anomalies of the Kidney and Urinary Tract (CAKUT), with a prevalence from 20 to 50%, but histological diagnoses are often lacking and the performed studies enrolled patients with a diagnosed cause [9,10].

The present study aims to epidemiologically analyze the causes of dialysis in a large cohort of patients aged less than 30 years whose data were retrieved from the Sicilian Registry of Nephrology, Dialysis, and Transplantation [11].

## 2. Materials and Methods

The study is in conformity with the guidelines of the Italian Data Protection Authority and in agreement with the Helsinki Declaration. Ethical approval was not necessary based on the Sicilian Registry of Nephrology, Dialysis and Transplantation, which is a collection of regional data, instituted by regional laws. The Sicilian Registry was initiated in 2008, with a decree (03423/08), to collect and analyze data. Informed consent is requested from all patients whose data are entered into the registry. However, as specified in Art. 1, no formal approval from ethical committees is needed to analyze data as they are made available only in anonymous form.

### 2.1. Population and Laboratory Data

In this retrospective cohort study, we recruited patients who started dialysis at an age less than 30 years old included in the Sicilian Registry of Nephrology, Dialysis and Transplantation from 1 January 2018 to 31 December 2020. Among a total of 6448 patients, 145 (2.24%) started dialysis treatment before the age of 30 years (mean age 22 ± 5 years, ranging from 3 to 30 years) in the considered period and were included in our database. About 49% (*n* = 71) of them started renal replacement therapy without a diagnosis and, among the 24 diabetic nephropathies, only 1 was histologically diagnosed.

In total, 59 patients were treated with various anti-hypertensive drugs (20 on monotherapy with angiotensin-converting enzyme (ACE) inhibitors, calcium channel blockers, α- and β-blockers, vasodilators, diuretics, or other drugs; 17 on double therapy; 12 on triple therapy; and 10 patients on quadruple or quintuple therapy with various combinations of these drugs). The main demographic, anthropometric, clinical and biochemical characteristics of the study population are detailed in Table 1.

Laboratory tests collected included serum phosphate, hemoglobin, C-reactive protein, iron, transferrin, ferritin, potassium, calcium, intact parathyroid hormone (PTH), albumin, glucose, triglycerides, cholesterol, bicarbonate, alkaline phosphatase, fractional urea clearance (Kt/V) and β2-microglobulin. Other information included pre-dialysis blood pressure levels, residual diuresis, comorbidities [dementia, hemiplegia, liver disease, history of arterial hypertension, vascular disease, chronic obstructive pulmonary disease (COPD) malignancy with/without metastasis, heart failure, psychiatric disease, dyslipidemia, prostatic hypertrophy] as well as pharmacological treatment such as anti-hypertensives (renin–angiotensin–aldosterone inhibitors [RAASIs], β-blockers), folic acid, calcium carbonate, cholecalciferol, insulin, aspirin, allopurinol, phosphorous binders, calcium mimetics, cortisone, erythropoiesis-stimulating factors (ESA), iron supplementation, immunosuppressive treatment, proton pump inhibitors, paricalcitol and vitamin B12. Details of this study are described elsewhere [11,12]. Laboratory and clinical data were collected locally from the register referents as part of the normal clinical practice, and then entered in the platform. Patients with histological diagnosis were included in group A (*n* = 74) whereas the others were included in group B (*n* = 71). Diabetic nephropathy without a histological diagnosis cannot be considered a specific diagnosis, due to the evidence in recent decades of non-diabetic nephropathies in diabetic patients (NDKD), as well as the hypertensive nephropathies that lacked biopsy.

### 2.2. Statistics

**Figure 1:**
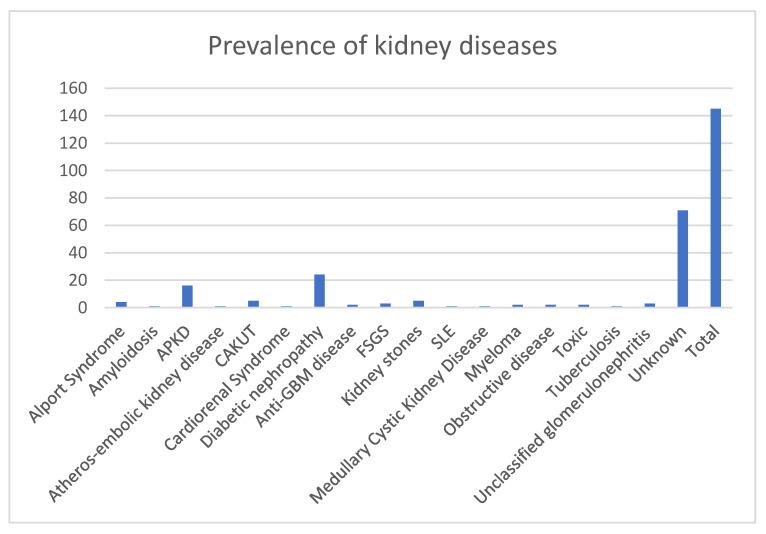
Prevalence of kidney diseases in patients who started dialysis before 30 years of age. Prevalence of diseases were Alport syndrome (*n* = 4, 2.76%), amyloidosis (*n* = 1, 0.69%), APKD (*n* = 16, 11.03%), atheros-embolic kidney disease (*n* = 1, 0.69%), CAKUT (*n* = 5, 3.45%), Cardiorenal Syndrome (*n* = 1, 0.69%), diabetic nephropathy (*n* = 24, 16.55%), Anti-GBM disease (*n* = 2, 1.38%), FSGS (*n* = 3, 2.07%), kidney stones (*n* = 5, 3.45%), LES (*n* = 1, 0.69%), Medullary Cystic Kidney Disease (*n* = 1, 0.69%), myeloma (*n* = 2, 1.38%), obstructive disease (*n* = 2, 1.38%), toxicity (*n* = 2, 1.38%), tuberculosis (b = 1, 0.69%), unclassified glomerulonephritis (*n* = 3, 2.07%) and unknown (*n* = 71, 48.96%). Sample size = 145. APKD = autosomal polycystic kidney diseases; CAKUT = Congenital Anomalies of the Kidney and Urinary Tract; Anti-GBM disease: anti-Glomerular Basement membrane disease; FSGS = focal and segmental glomerulosclerosis; SLE = systemic lupus erythematosus.

Causes of dialysis initiation were reported as frequency and percentage (Figure 1). The whole sample was divided into two groups, according to the presence or absence of a diagnosis for CKD.

The number of missing data varied across variables. In detail, albumin was missing in about 48% of measures, potassium in 47%, calcium in 40%, body mass index (BMI) in 33%, PTH in 50%, ferritin in 61%, C-reactive protein (CRP) in 62%, HCO_3_ in 74% and systolic blood pressure in 12%. Other variables included in the multivariate models had less than 10% of missing data. Missing values were neither related to the center which provided the data nor to specific characteristics of the patients, so were considered completely at random. No laboratory data were imputed, as they were not clinically related to our outcomes. RAASI intake and systolic pressure were imputed using the median value. Hypertensive nephropathy (*n* = 18) was included as an unknown diagnosis, due to the absence of histological detection. Baseline features were reported as mean ± standard deviation, median [IQR] or *n* (%) according to their distribution and were compared through a Student *T*-test, Mann–Whitney test or Pearson Chi-Square test, as appropriate. Logistic regression and ROC analysis were performed using the absence of diagnosis as a dependent variable. *p*-values < 0.05 were considered statistically significant. *p*-values < 0.2 were considered as possible confounding variables, to reduce the estimation bias. All confounding variables were added in the multivariate model (RAASI intake and systolic pressure), and the predicted value of the multivariate logistic regression was used as an independent variable in ROC analysis. Statistical analysis was performed using SPSS version 24 (version 24.0; IBM Corporation, Armonk, NY, USA), MedCalc version 20 64-bit (MedCalc software Ltd., Ostend, Belgium) and STATA version 13 (Stata Corporation, College Station, TX, USA).

## 3. Results

A total of 145 patients, who started dialysis before 30 years of age, were included in the analysis. Primary renal diseases which caused ERSD were detected in 74 patients. Among these genetic diseases caused by ESRD in 23 patients [autosomal polycystic kidney diseases (APKDs) = 16, Alport syndrome = 4, anti-Glomerular Basement membrane disease (Anti-GBM disease) = 2 and Medullary Cystic Kidney Disease Type I = 1]. We were not able to include the five Congenital Anomalies of the Kidney and Urinary Tract (CAKUT) among the genetic diseases due to the missing genetic details. Only one case of systemic lupus erythematosus (SLE) and three not-specified glomerulonephritis (GMN) cases accounted for dialysis treatments in our analysis (Figure 1); 71 patients started dialysis without a diagnosis of primary renal disease.

The mean age for the whole sample was 22 ± 6 years; 11 were diabetic, and a history of arterial hypertension was known in 59 patients. The clinical, demographic and anthropometric characteristics of the whole population are shown in Table 1.

Analyzing the differences between patients with diagnosed renal diseases and with unknown renal diseases, RAASI intake was different between the two groups (4% vs. 15%, *p* = 0.02) and systolic blood pressure had a trend of significance (140 [132–146] vs. 144 [130–158], *p* = 0.18) without reaching statistical significance. These two will be investigated as confounding factors (Table 1). To confirm the differences in systolic pressures between the two groups, the standardized mean difference and standardized deviation ratio were 0.83 and 1.53, respectively.

**Figure 2:**
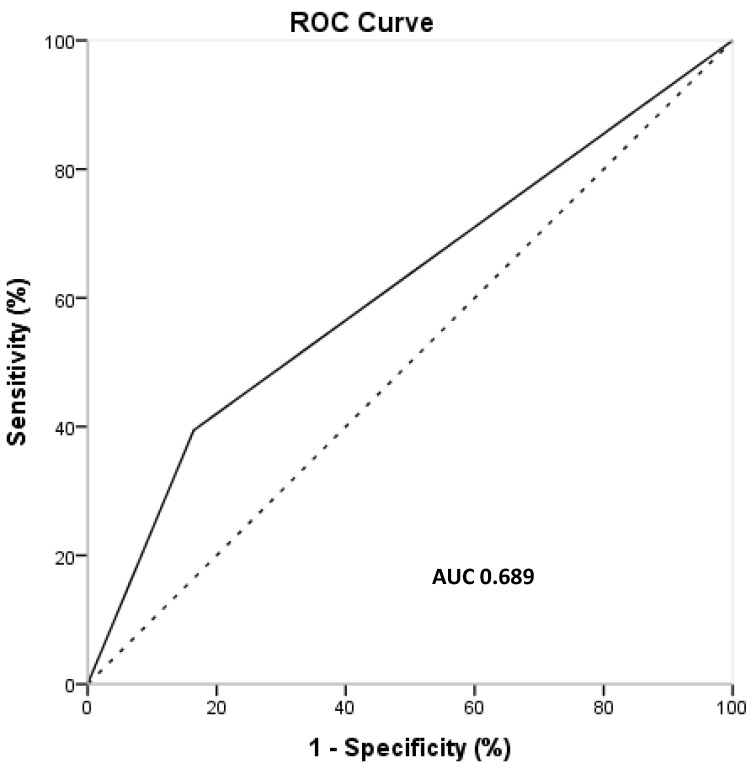
ROC curve using unknown diagnosis as the dependent variable and the predictor value of a multivariate model including angiotensin-conversion enzyme inhibitor consumption and systolic pressure.

The logistic regression using an unknown diagnosis as the dependent variable showed an OR of 1.03 (95% CI 0.996/1.059, *p* = 0.083) for systolic blood pressure and an OR of 3.41 (95% CI 1.24/11.29, *p* = 0.046) for RAASI therapy. No other association with *p* < 0.2 were found. ROC curve analysis, adjusted for systolic blood pressure and RAASI treatment, provided an AUC of 0.689 (Figure 2). In keeping with these results, about 69% of the unknown causes of dialysis could be explained by our model which includes systolic pressure and RAASI use.

## 4. Discussion

Our epidemiological analysis showed that diabetes mellitus and autosomal polycystic kidney disease (APKD) were the most prevalent causes of dialysis in patients who started dialysis before 30 years of age and that RAASI intake and systolic blood pressure were related to unknown diagnosis. However, the diagnosis of about half of the cases of early dialysis was unknown, lacking a renal biopsy, and this could cause significant differences between our results and the literature.

Although Roselbum et al. [9] reported that CAKUTs play a causative role in about 30–50% of pediatric CKDs, dialysis initiation in our sample was caused by CAKUTs in only 3.45% of patients. Similarly, the retrospective analysis conducted by Okuda Y. et al. [10] showed that 19% of the need for dialysis in patients aged less than 21 years old was caused by CAKUTs. The differences between our sample and the previously cited article could be due to either the higher rate of diagnosis or the different examined populations that were not detected.

In comparing patients having a histological diagnosis with patients with an unknown diagnosis, this study highlighted that arterial blood pressure was higher and the use of RAASIs was more prevalent in patients without a renal histological diagnosis. Furthermore, both of them were related to the absence of diagnosis in the logistic regression analysis. Hypertensive nephropathy is due to increased RAAS activation, epithelial–mesenchymal transition (EMT) and tubulointerstitial fibrosis. It starts with microvascular and post-glomerular peritubular damage, leading to podocyte injury [13]. Consequently, reduced kidney dimensions, worse cortical differentiation and progressive glomerular sclerosis that reduces kidney function were experienced [14]. Although arterial hypertension is among the major causes of CKD and increases the disease progression risk [15], its impact slowly damages kidney function, mostly in adult patients. For this reason, the high prevalence found in our sample disagreed with the epidemiological evaluation in the literature on child and young patients evaluated by histological analysis. For example, in a Serbian prospective observation study [16], the most common histopathological diagnoses were focal segmental glomerulosclerosis, mesangioproliferative GN, IgA nephropathy, minimal change disease, lupus nephritis and Henoch–Schönlein nephritis. No hypertensive diseases were biopsied in this study and no hypertensive nephropathy was diagnosed. According to the 2021 KDIGO guidelines, only protein–creatinine ratio (PCR) monitoring is suggested for biopsy indication. Similarly, Japanese practice guidelines [17] also include steroid-resistant or congenital nephrotic syndrome, proteinuria higher than 0.5 g/g Cr and systemic disease as indications for kidney biopsy, but arterial hypertension without proteinuria is not considered a biopsy indication. Although arterial hypertension has not been included among the reasons for performing biopsy [18], our study highlighted that a high prevalence of hypertensive nephropathy was detected in dialyzed young patients without a histologic diagnosis.

In keeping with KDIGO 2022, arterial pressure should be treated in children affected by CKD if this is higher than 50th percentile for age and gender, and the first-line therapy includes RAASIs. Furthermore, proteinuria in children is often treated by administering RAASIs, without histologic diagnosis. This therapy can hide nephrotic syndrome, reducing proteinuria and arterial hypertension. As a consequence, this avoids renal biopsy without an efficient treatment of underlying glomerulonephritis.

In the past, albuminuria in diabetic patients was managed as a factor in the pathophysiological progression of diabetic nephropathy. This idea was changed after high rates of various glomerulonephritis were found in diabetic patients. As reported by Anders HJ et al. [19], histologic diagnosis in diabetic patients was often different from diabetic nephropathy. In detail, diabetic nephropathy was diagnosed in less than 40% [20] of the kidney biopsies in diabetic subjects and, among the non-diabetic kidney diseases (NDKDs), the most prevalent were focal segmental glomerulosclerosis, hypertensive nephrosclerosis, acute tubular necrosis (ATN), IgA nephropathy and membranous GN.

Similar results have been found in a metanalysis conducted on 4876 biopsies on diabetic patients [21]. In our sample, only 1 diabetic nephropathy was histologically identified among a total of 24 diagnoses of diabetic nephropathy, and other NDKDs were unacknowledged. According to our hypothesis, whilst proteinuria in diabetic patients was standardly considered as a sign of diabetic nephropathy development, its management is now improved by biopsy.

Although the morphology of kidneys can suggest the nature of kidney damage, and ultrasonography could help us to discriminate between glomerulonephritis and hypertensive nephropathy [22], the gold standard is renal biopsy. Moreover, neither elastography is able to evaluate the degree of fibrosis detected by kidney biopsy, and it cannot be considered a good method to discriminate hypertensive nephropathy from other forms of glomerulonephritis [23]. Indeed, conversely to the larger epidemiological study that showed a prevalence of about 1.4% of renal biopsies in patients affected by arterial hypertension [24], independently of age, we reported a prevalence of about 12% in children. Consequently, we can suppose that a higher proportion of them were erroneously classified as having hypertensive nephropathy due to the missing renal biopsy. Although renal biopsy could carry significant risk, it could improve the diagnostic specificity and discriminate against those cases in which hypertension is not the cause but a consequence. Thus, the management and treatment could be early improved.

Our study has some limitations. The main limitations of our study include observational design and, thus, the possibility of residual confounding. Furthermore, our analysis included only young patients who descended from a single Italian region, thus reducing the generalizability of our study. Furthermore, only ten patients died in that period, six from diagnosed causes and four from an unknown cause. This number of cases is too low to perform strong statistical analysis. Thus, mortality analysis was not performed. Another limitation is the small sample size and missing data. Indeed, considering our different prevalence of RAASI intake (4% vs. 15%), our analysis had a power of 65%.

## 5. Conclusions

In conclusion, according to our results, young patients with hypertension or treated with RAASIs were more likely to not have kidney biopsy and histological diagnosis, starting dialysis without a specific diagnosis. Thus, patients who manifested worsening renal function markers and were affected by arterial hypertension or treated with RAASI seem to need more in-depth diagnostic evaluations. According to this, renal biopsy should be kept in mind in cases where there is no complete response to the treatment. Furthermore, other larger studies should be carried out to confirm our results, which can improve the management of renal diseases in young patients Thus, we suggest that a kidney biopsy should be performed on these young patients, in addition to the clinical and laboratory assessment.

## Figures and Tables

**Table 1:** Baseline differences between diagnosed and unknown renal disease groups.

	Diagnosed (*n* = 74)	Unknown (*n* = 71)	*p*
BMI (kg/m^2^)	20.9 ± 5.0	22.1 ± 4.9	0.26
Hemoglobin (g/dL)	10.3 ± 1.3	10.1 ± 1.4	0.51
Albumin (g/dL)	3.8 [3.4–4.1]	3.8 [3.5–4.0]	0.81
Triglycerides (mg/dL)	121 [77–172]	93 [79–170]	0.44
HDL (mg/dL)	10.2 [9.2–11.1]	10.2 [9.4–11.0]	0.56
**Systolic blood pressure (mmHg)**	**140 [132–146]**	**144 [130–158]**	**0.18**
Age (years)	24 [20–27]	23 [18–27]	0.74
TSAT (%)	17 [13–36]	25 [13–44]	0.83
PTH (pg/mL)	253 [157–450]	258 [170–621]	0.66
Serum phosphate (mg/dL)	5.5 [4.8–6.3]	4.9 [4.5–6.4]	0.30
Potassium (mmol/L)	4.9 [4.4–5.4]	4.7 [4.3–5.3]	0.74
**RAASIs, *n* (%)**	**3 (4)**	**11 (15)**	**0.02**
Connective disease, *n* (%)	3 (4)	1 (1)	0.35
Heart failure, *n* (%)	0 (0)	2 (3)	0.49
Arrhythmia, *n* (%)	1 (1)	3 (4)	0.62
Thyroid diseases, *n* (%)	4 (5)	4 (5)	1.00
History of Diabetes, *n* (%)	5 (7)	6 (8)	0.76
Liver disease, *n* (%)	1 (1)	2 (3)	1.00
IBD, *n* (%)	1 (1)	1 (1)	1.00

BMI = body mass index; HDL = high-density level; TSAT = transferrin saturation; PTH = parathormone; RAASIs = renin-angiotensin-aldosterone system inhibitors IBD = inflammatory bowel disease.

## Data Availability

The data were retrieved from the Sicilian Registry of Nephrology, Dialysis, and Transplantation (http://www.crtsicilia.it/PUBLIC/RegistroRSNDT/CentriDialisiETx.aspx (accessed on 6 July 2022)).
